# Supplementary figures and images for: Chronic cholecystitis: Diagnostic and therapeutic insights from formerly bile-farmed Asiatic black bears (Ursus thibetanus)
Source: PLoS One. 2022 Mar 3;17(3):e0264391. doi: 10.1371/journal.pone.0264391 (PMC8893648; doi:10.1371/journal.pone.0264391)

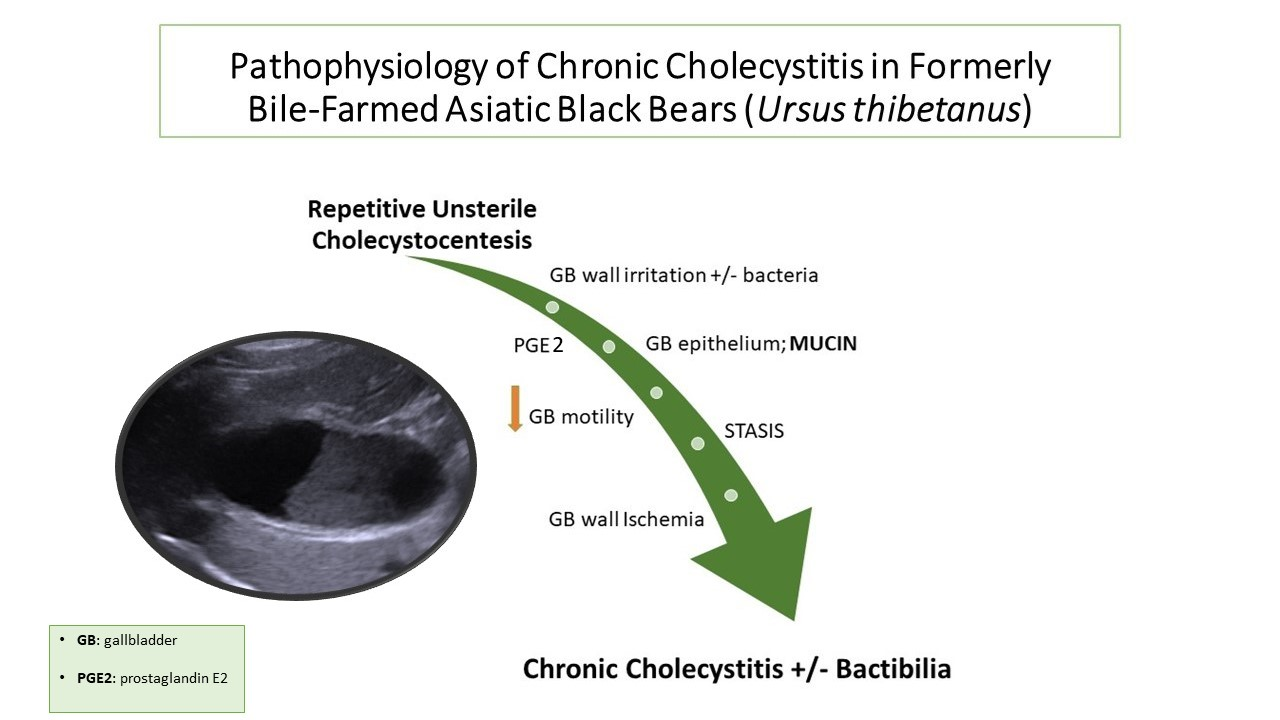

Supplement: S1 Fig — (TIF) [file pone.0264391.s002.tif]

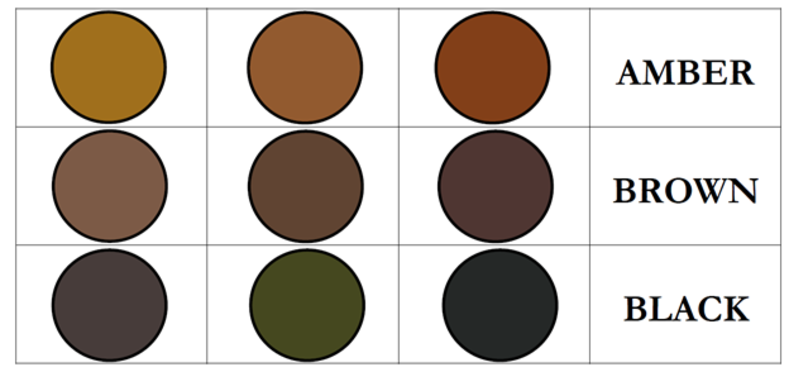

Supplement: S2 Fig — The color samples were obtained from photographs of the collected bile samples. (TIF) [file pone.0264391.s003.tif]

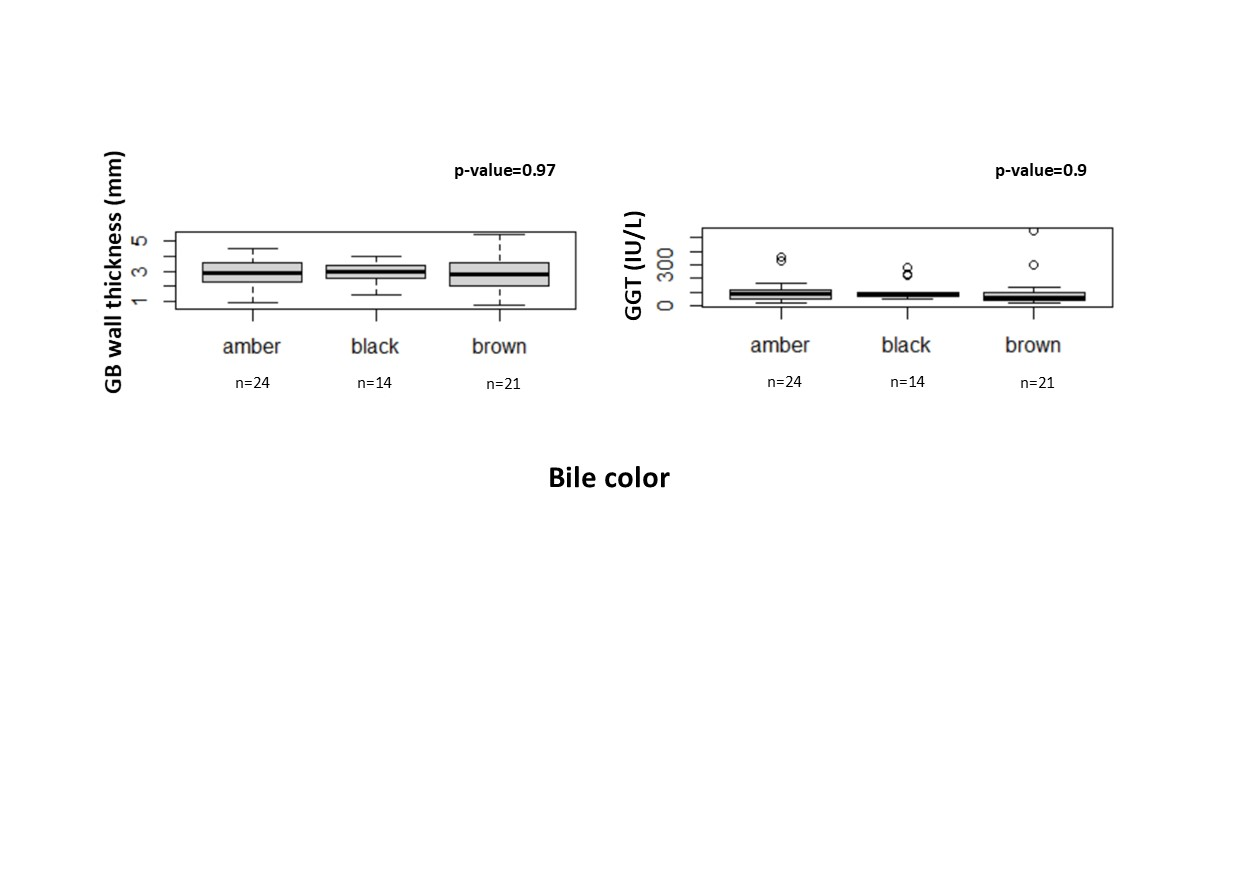

Supplement: S3 Fig — Gallbladder wall thickness and GGT means were compared between different bile color categories of Asiatic black bears (number of Asiatic black bears in each group is depicted) with chronic cholecystitis. The comparisons (one-way ANOVA) were found to be statistically insignificant with p-value> 0.05. (TIF) [file pone.0264391.s004.tif]

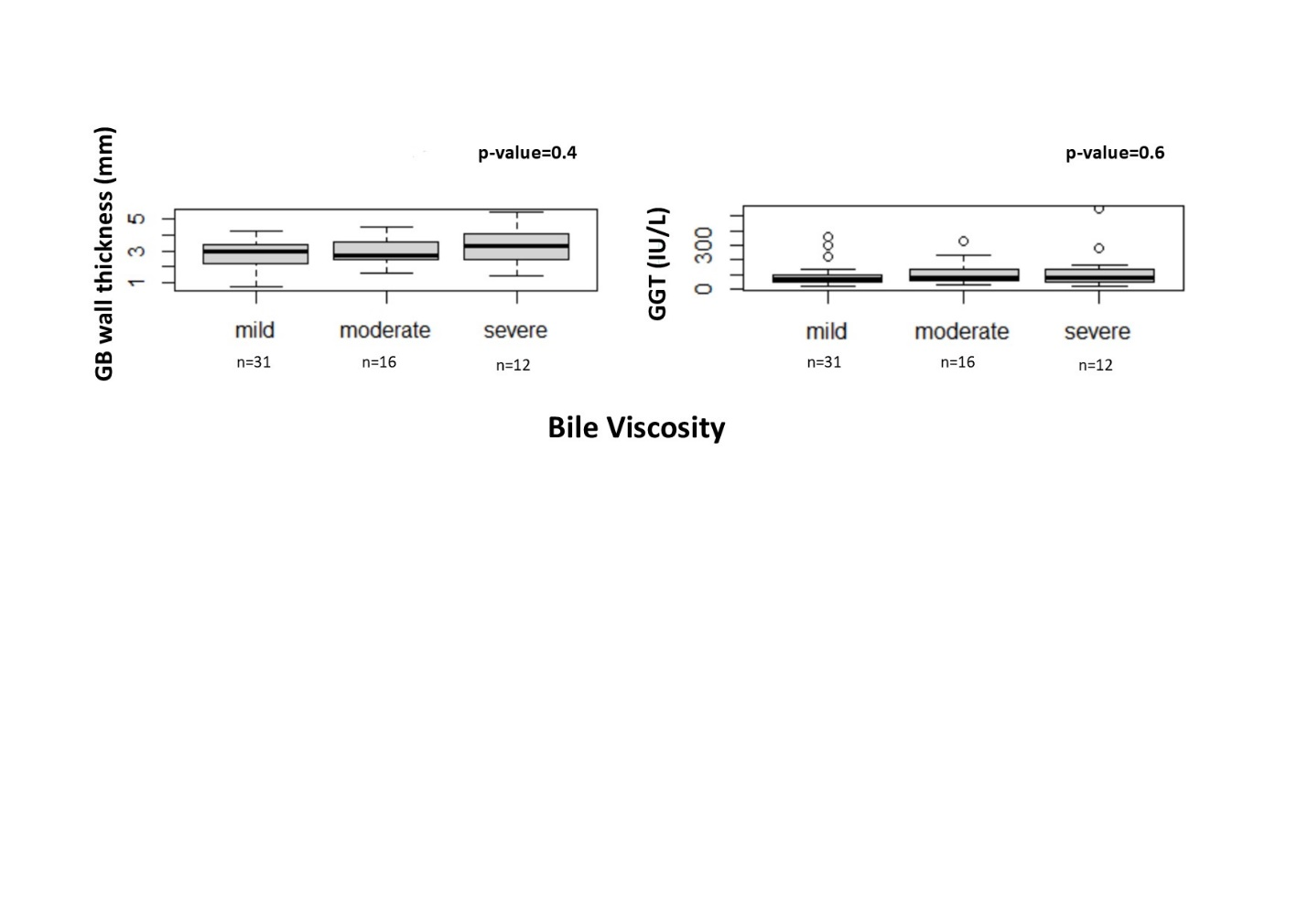

Supplement: S4 Fig — Gallbladder wall thickness and GGT means were compared between different bile viscosity levels of Asiatic black bears (number of Asiatic black bears in each group is depicted) with chronic cholecystitis. The comparisons (one-way ANOVA) were found to be statistically insignificant with p-value> 0.05. (TIF) [file pone.0264391.s005.tif]

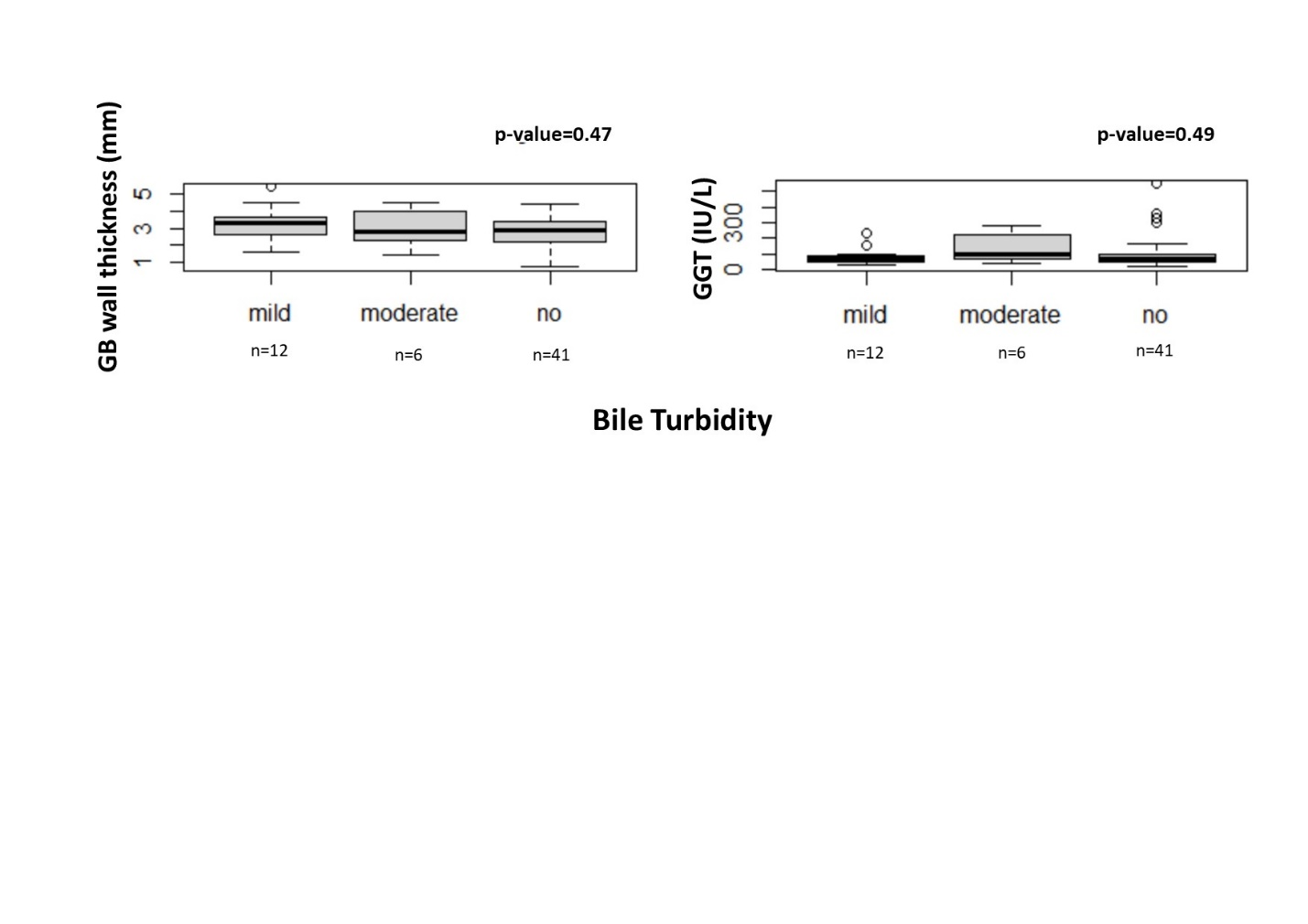

Supplement: S5 Fig — Gallbladder wall thickness and GGT means were compared between different bile turbidity levels of Asiatic black bears (number of Asiatic black bears in each group is depicted) with chronic cholecystitis. The comparisons (one-way ANOVA) were found to be statistically insignificant with p-value> 0.05. (TIF) [file pone.0264391.s006.tif]

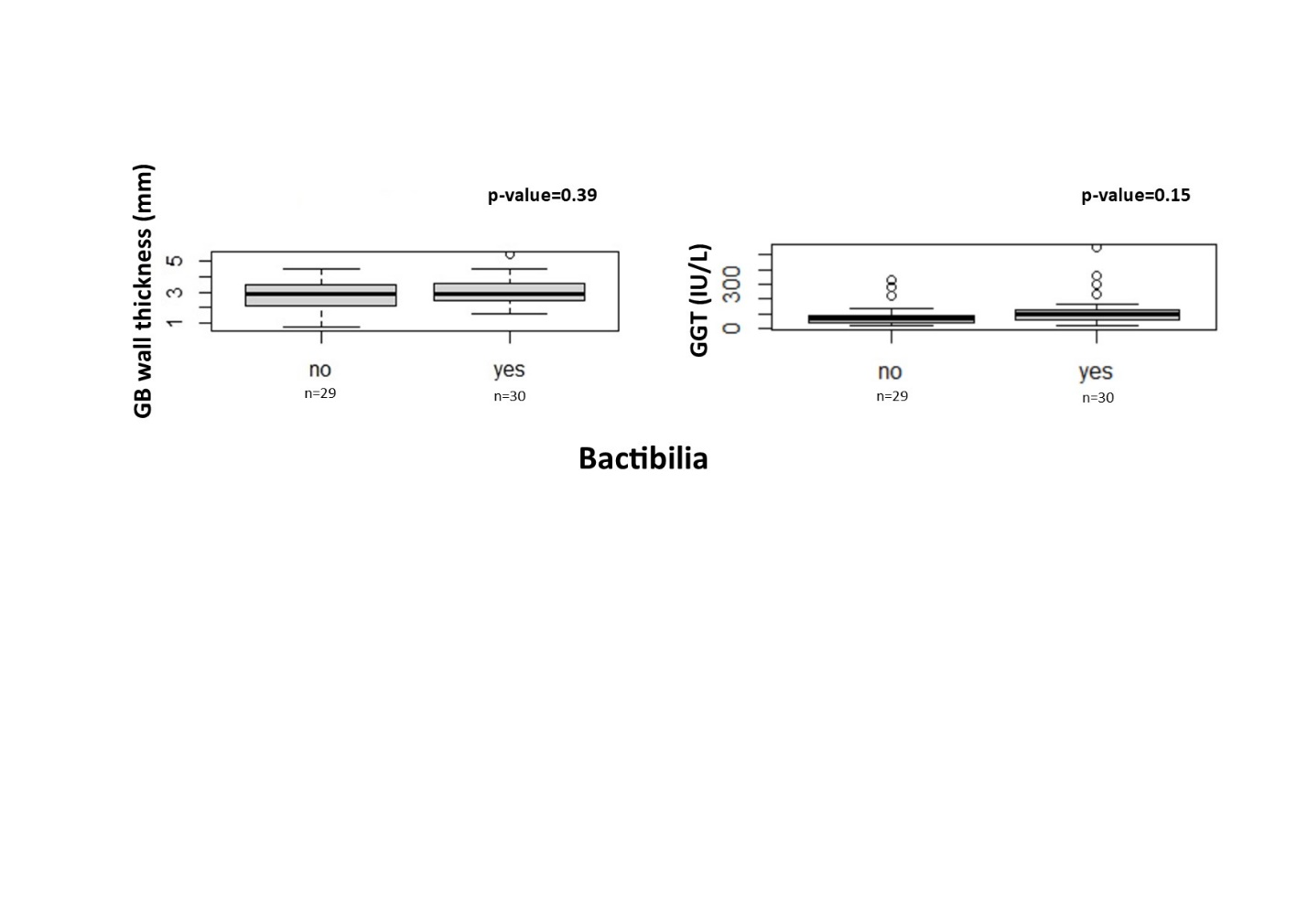

Supplement: S6 Fig — Gallbladder wall thickness and GGT means were compared between positive and negative bile cultures of Asiatic black bears (number of Asiatic black bears in each group is depicted) with chronic cholecystitis. The comparisons (one-way ANOVA) were found to be statistically insignificant with p-value> 0.05. (TIF) [file pone.0264391.s007.tif]
