# Supplementary material for: Chronic cholecystitis: Diagnostic and therapeutic insights from formerly bile-farmed Asiatic black bears (Ursus thibetanus)
Source: PLoS One. 2022 Mar 3;17(3):e0264391. doi: 10.1371/journal.pone.0264391 (PMC8893648; doi:10.1371/journal.pone.0264391)
Supplement: Attachment — Submitted filename: Response to Reviewers.docx [file pone.0264391.s008.docx]

**Rebuttal letter points – PONE-D-21-26096**

**Dear Academic Editor, dear Reviewer,**

Thank you very much for considering our publication “Cholecystocentesis: a valuable diagnostic tool for successful treatment of chronic cholecystitis in formerly bile-farmed Asiatic black bears (*Ursus thibetanus*)”to be reviewed for PLOS ONE. We hope that the new version of our manuscript fulfills the publication requirements of your journal. Please find below the reply to each point raised in the comments. Please also find uploaded a marked-up copy of our revised manuscript entitled “Revised Manuscript with Track Changes”, an unmarked version of our revised paper entitled “Manuscript”, the formatted figures and the supporting information files.

**Academic Editor’s comments:**

1. Revision of the abstract to highlight the significance and strength of the research. 🡪 Answer (A): the changes were done accordingly.
2. Revision of the text (discussion) to highlight the significant and strength of the research. 🡪 A: the changes were done accordingly.
3. Provision of potential reviewers. 🡪 A: we would like to suggest the following researchers as potential reviewers:
   a. **Jan S Suchodolski**

[<https://orcid.org/0000-0002-2176-6932> orcid.org/0000-0002-2176-6932](https://orcid.org/0000-0002-2176-6932)

**Email: jsuchodolski@cvm.tamu.edu**
Texas A&M University College Station
UNITED STATES
**Sections:** Veterinary science
**Keywords:** Biology and life sciences, Microbial ecology, Immunology, Inflammation, Immunity, Clinical immunology, Microbiology, Microbial pathogens, Veterinary science, Veterinary diseases, Veterinary medicine, Small animal care, Veterinary diagnostics, Veterinary microbiology, Biochemistry, Enzymes, Medicine and health sciences, Gastroenterology and hepatology, Infectious diseases, Bacterial diseases, Zoonoses, Leishmaniasis, Pathology and laboratory medicine, Anatomical pathology, Histopathology, Endoscopy, Hepatosplenomegaly, Diagnostic medicine, Retrospective studies, Clinical research design

b. **Kristin Mühldorfer**

[<https://orcid.org/0000-0001-8023-4736> orcid.org/0000-0001-8023-4736](https://orcid.org/0000-0001-8023-4736)

**Email:** [muehldorfer@izw-berlin.de](mailto:muehldorfer@izw-berlin.de)

Leibniz Institute for Zoo and Wildlife Research (IZW)
GERMANY
**Sections:** Veterinary science, Microbiology - Bacteriology, Infectious diseases - Bacterial and fungal diseases
**Keywords:** Biology and life sciences, Microbial evolution, Bacterial evolution, Microbial genomics, Bacterial genomics, Bacterial genomes, Microbiome, Bacterial genetics, Microbiology, Bacteriology, Bacterial taxonomy, Gram negative bacteria, Gram positive bacteria, Fungal pathogens, Molecular biology, Molecular biology techniques, Mycology, Veterinary parasitology, Veterinary science, Veterinary diseases, Veterinary medicine, Veterinary diagnostics, Veterinary microbiology, Veterinary pathology, Veterinary virology, Zoonoses, Research and analysis methods

c. **Peter Starkel**

**Email:** [peter.starkel@uclouvain.be](mailto:peter.starkel@uclouvain.be)
Cliniques Universitaires Saint-Luc
BELGIUM
**Sections:** Gastroenterology and hepatology
**Keywords:** Signal transduction, Signaling cascades, AKT signaling cascade, Primary biliary cirrhosis, Crohn's disease, Medicine and health sciences, Gastroenterology and hepatology, Inflammatory bowel disease, Biliary disorders, Primary sclerosing cholangitis, Hepatitis

d. **Maite Garcia Fernández-Barrena**

[<https://orcid.org/0000-0003-0375-6236> orcid.org/0000-0003-0375-6236](https://orcid.org/0000-0003-0375-6236)

**Email:** [magarfer@unav.es](mailto:magarfer@unav.es)
Centre for Applied Medical Research, University of Navarra
SPAIN
**Sections:** Gastroenterology and hepatology, Cancer - Genetics and screening, Genetics - Gene expression; Epigenetics; Alternative splicing; RNA splicing; Molecular genetics
**Keywords:** Biology and life sciences, Biochemistry, Medicine and health sciences, Gastroenterology and hepatology, Inflammatory bowel disease, Hepatocellular

4. Please ensure that your manuscript meets PLOS ONE's style requirements, including those for file naming 🡪 A: The manuscript including the file names was formatted according to the PLOS ONE style requirements.
5. Upload minimal dataset underlying the results- as supporting information file. 🡪 A: We added the minimal dataset.
6. Include ethics statement in the material and methods: 🡪 A: an ethical statement was given in line 95-99.

**Reviewer #1 comments:**

Thank you very much for taking the time and reviewing our manuscript. Your input is very much appreciated! Please find below our replies to your points:

1. Manuscript overloaded with illustrative material: 🡪 A: We deleted 5 figures and moved them to the supportive information section. Please let us know in case the manuscript still appears overloaded and would like us to delete additional figures. Boxes and whiskers were explained in the legends.
2. Clarification of manuscript’s fundamental scientific importance. 🡪 A: We consider our findings of fundamental scientific importance, since they provide novel and comprehensive information for the diagnosis and treatment of hepatobiliary disease in formerly bile farmed bears, an understudied topic of great concern for wildlife veterinarians, conservationists and bear husbandry related professionals in South East Asia and China. Bear bile farming is still a common practice in Asia, despite being the main threat for population decline of Asiatic black bears and compromising the welfare of at least 17000 individuals, making this manuscript an essential contribution. Moreover, the findings presented in the current study may be applicable in other mammalian species and are not limited only to Asiatic black bears.
3. Provision of description of GGT as an assay: 🡪 A: description of the assay was given in line 271.
4. Lines 258-260: "Furthermore, the comparison of gallbladder wall thickness and GGT means between bacteria positive and negative bile cultures was insignificant ...". Was the "comparison" really insignificant? May be difference was? 🡪 A: The comparison of GGT and gallbladder wall thickness was statistically insignificant between bacteria positive and bacteria negative bile samples collected from individuals with chronic cholecystitis and both p-values were greater than 0.05. The clarification can be found in the lines 235-238 of the revised manuscript.
5. Clear explanation of treatment efficacy estimation (lines 285-286, non-revised MS version)🡪A: The clarification of treatment efficacy estimation is given in lines 259-261.

Other corrections

1. The email of the corresponding author was corrected in line 22.
2. The dose of the continuous rate infusion of propofol was corrected in lines 123-125.
3. Escherichia coli was added as one of the most prevalent bacterial genera identified in the bile cultures in line 235.
4. The supportive information captions were added in lines 519-533.

Thank you very much for re-considering our manuscript to be published after major revision.

Kind regards,

Szilvia K. Kalogeropoulu
