# Supplementary material for: Chronic cholecystitis: Diagnostic and therapeutic insights from formerly bile-farmed Asiatic black bears (Ursus thibetanus)
Source: PLoS One. 2022 Mar 3;17(3):e0264391. doi: 10.1371/journal.pone.0264391 (PMC8893648; doi:10.1371/journal.pone.0264391)
Supplement: Attachment — Submitted filename: Response to Reviewers.docx [file pone.0264391.s009.docx]

**Rebuttal letter points – PONE-D-21-26096R1**

**Dear Academic Editor, dear Reviewer,**

Thank you very much for taking the time and reviewing our manuscript after the first major revision. We hope that this edited version following your recommendations and comments will fulfill the requirements for publication in PLOS ONE. Please see below the reply to each point raised in the comments. Please also find attached a marked-up copy of our revised manuscript entitled “Revised Manuscript with Track Changes”, an unmarked version of our revised paper entitled “Manuscript”, the formatted figures and the supporting information files.

**Academic editor’s comments:**

The comments of the reviewer were considered carefully and effort was made to answer every point precisely and modify the text accordingly.

**Reviewer comments:**

Thank you again for meticulously reviewing our manuscript and helping us to present and communicate better our findings. Your comments truly improved the quality of this manuscript!

1. Title correction and making it more neutral**🡪Answer (A):** the manuscript’s current title is “Cholecystocentesis: a valuable diagnostic tool for successful treatment of chronic cholecystitis in formerly bile-farmed Asiatic black bears (Ursus thibetanus)”. Our suggested new title is: “Chronic cholecystitis: diagnostic and therapeutic insights from formerly bile-farmed Asiatic black bears (*Ursus thibetanus*)”. Please find it in lines: 1-3.
2. Provision of description of GGT as an assay**🡪A:** Please find GGT assay description in lines 116-117. The GGT analysis was done with the Vettest 8008 machine from IDEXX. The machine uses dry-slide technology (inputted in line 114) and GGT was detected and quantified with colorimetric analysis. We are hoping that our new explanation sufficiently answered your question.
3. How two different parameters - gallbladder bile turbidity and color can be statistically "compared"? To my mind one can analyse either correlation between two quantitative parameters, or qualitative effects of independent variable on dependent one. Respective figure (fig.2) does not clarify this question. If the turbidity was classified on three categories "mild", "moderate", "no", what do the intermediate points (0.5, 1.5) on Y-axe means? Additionally, this figure is too flattened to be perceived correctly. Three mentions on statistical significance of the comparison (in text, in caption and on the diagram) seem to be redundant. Instead, authors should explain the box and whiskers values**🡪 A:** Gallbladder bile turbidity and color have been treated as categorical variables with three levels each. A chi-square test can be used to analyze two categorical variables and evaluates whether there is a significant association between the categories of the variables. Usually, box plots help to visualize the distribution of quantitative values in a field. It is recognized that in our case that we have two categorical variables the box plot is not an appropriate visualization method. Moreover, in Figure 2, the categories of turbidity “non-turbid”, “mild” and “moderate” were replaced by “0”, “1” and “2” respectively to allow the visualization of the data as a box-plot and the intermittent points 0.5 and 1.5 did not represent any category of bile turbidity. The Pearson’s Chi- squared test was repeated and followed by a Bonferroni post-hoc analysis for groupwise comparison to identify which categorical pair/s of the variables analyzed was/were significantly associated. The significant p-value of the chi-square test is attributed to the negative association between “black” bile color and “non-turbid” turbidity. Regarding result visualization for small data sets and categorical variables a bar plot is recommended. Despite the statistically significant relationship between “black” and “non-turbid”, this finding was poorly visualized by the bar plot. Bar plots are a descriptive tool that show the distribution of the data and since “amber” was the most common finding led to its “overrepresentation” in the graph suggesting that the relationship with “non-turbid” is statistically significant, while the association of “black” and “non-turbid” appears to be insignificant (please see below). Considering the above points and trying to provide clarity for the reader we would like to remove the former box plot (fig 2) and present our findings only via text. Changes in the “materials and methods” section can be found in lines 195-198. Modifications in the results and discussion are made in lines 212-214 and 296-297 respectively. Thank you for your critical thought, we hope that the statistical changes are conform with you. We believe that your comment has much improved our result communication to the reader!

4. What is the total time animal spent under anesthesia? **🡪 A:** Data were collected during essential medical interventions suited to each bear’s medical needs and were not part of a designed experiment, hence the total duration of anesthesia varied between individuals. In an effort to standardize sampling, blood samples were collected within 20-40 minutes post darting and bile samples between 40-60 minutes post darting. This information was added to the manuscript text in lines 112 and 115 respectively.
5. "Bears with positive bile cultures were treated based on antibiogram or received other antibiotics in cases of bacterial resistance for 14 or 30 days". If I understand correctly, the exact course of antibiotic treatment was chosen personally, basing of the individual susceptibility to different antibiotics. How can the durations and effectiveness of the courses can be compared and optimal duration established? For me and other readers who are not experts in veterinary the optimal duration of the course seems to be strongly depended on the infection agent and medicines applied. **🡪A:** The exact course of antibiotic treatment was not chosen personally, but according to the existing literature for the treatment of bacterial cholecystitis. From both human and veterinary medicine there are antibiotic treatment recommendations (agent, treatment duration) against specific microorganisms causing cholecystitis. In cases where bears were resistant to the antibiotics tested in the antibiogram, an antibiotic (not included in the antibiogram) was chosen based on the existing treatment guidelines from other species. The clarification was made in lines 128-133. Literature examples of treatment recommendations were cited and the reference list numbering was adjusted accordingly.
6. Omit Figure 1**🡪A:** Figure 1 was omitted and added to supporting information. Figure numbering and naming was changed accordingly. Additionally, the legend of Figure 1 as a supporting figure was given in lines 534-535.

Thank you very much considering our manuscript for publication after a second major revision.

Kind Regards,

Szilvia K. Kalogeropoulu
